# Bridging the gap: From clinical intuition to structured cutaneous squamous cell carcinoma classification

**DOI:** 10.1111/jdv.20548

**Published:** 2025-02-25

**Authors:** Christoffer Gebhardt

**Affiliations:** ^1^ Department of Dermatology and Venereology, University Skin Cancer Center Hamburg University Medical Center Hamburg‐Eppendorf (UKE) Hamburg Germany; ^2^ Fleur Hiege Center for Skin Cancer Research University Medical Center Hamburg‐Eppendorf (UKE) Hamburg Germany

The paper ‘Operational classification of cutaneous squamous cell carcinomas based on unsupervised clustering of real cases by experts’ by Gaudy‐Marqueste presents a groundbreaking approach to classifying cutaneous squamous cell carcinoma (cSCC), addressing critical shortcomings in existing staging systems.^1^ This innovative methodology has important implications for enhancing clinical decision‐making and refining the design of future clinical research.

The study's approach of using unsupervised clustering of real cSCC cases by a group of 18 international specialists from different relevant disciplines, enhancing its validity and applicability, is particularly noteworthy. By tapping into the collective unconscious expertise of clinicians, the researchers have developed a classification system that is inherently aligned with real‐world clinical decision‐making processes. This approach contrasts with traditional staging systems that are often based on specific tumour characteristics and risk factors.^1^

The resulting six‐group classification system—comprising easy‐to‐treat, complex‐to‐treat due to tumour and/or patient characteristics, multiple tumours, locally advanced without regional metastases, regional metastases and visceral metastases—offers a practical framework for clinicians. This structure can help streamline tumour board discussions and facilitate more consistent decision‐making across different healthcare settings. The high concordance (94%) in case allocation between new practitioners and experts demonstrates the classification's intuitive nature and potential for widespread adoption.^1^

One of the most significant contributions of this classification system is its potential impact on clinical trial design. By providing a method to create more homogeneous patient groups, it addresses a longstanding challenge in cSCC research. This could lead to more targeted and efficient clinical trials, potentially accelerating the development of new treatments and improving patient outcomes.^2^

The study's success in finding a mathematical consensus, resulting in a five‐cluster classification of ‘difficult‐to‐treat’ cases, despite the high heterogeneity of cSCC cases is remarkable. This achievement suggests that the classification system captures fundamental aspects of cSCC presentation and behaviour that are relevant across diverse patient populations. Such a universally applicable system could greatly enhance global collaboration in cSCC research and treatment.

Importantly, this new classification system is designed to complement, rather than replace, existing staging systems like the American Joint Committee on Cancer (AJCC) and Brigham and Women's Hospital (BWH) systems. For instance, while AJCC 8 has limitations in predicting poor outcomes, and BWH demonstrates better positive predictive value,^3^ neither system incorporates broader clinical complexity as effectively as this new classification. While these traditional systems focus on specific tumour characteristics for prognostication, the new classification offers a more clinically oriented approach based on expert consensus. This complementary nature allows for a more comprehensive assessment of cSCC cases, potentially improving both prognostication and treatment decision‐making and should therefore be implemented into international guideline recommendations.^4^, ^5^, ^6^

In conclusion, this paper presents a significant advancement in cSCC classification that has the potential to improve both clinical practice and research. By bridging the gap between intuitive clinical decision‐making and structured classification systems, it offers a valuable, intuitive and easily applicable tool for oncologists, dermatologists and researchers alike. As cSCC continues to be a significant health concern worldwide, such innovations in classification and management are crucial for improving patient care and outcomes.

## CONFLICT OF INTEREST STATEMENT

C.G. is on the advisory board or has received honoraria from Almirall, Amgen, Beiersdorf, BioNTech, Bristol‐Myers Squibb, Delcath, Immunocore, Janssen, Medscape, MSD Sharp & Dohme, Novartis, Onkowissen, Pierre‐Fabre Pharma, Roche, Sanofi Genzyme, SUN Pharma and Sysmex, research funding from Novartis, Regeneron and Sanofi Genzyme, and travel support from Bristol‐Myers Squibb, Pierre Fabre Pharma and SUN Pharma, outside the submitted work. C.G. is co‐founder of Dermagnostix and Dermagnostix R&D.

## Data Availability

Data sharing not applicable to this article as no datasets were generated or analysed during the current study.
